# Mental Health Symptoms Unexpectedly Increased in Students Aged 11–19 Years During the 3.5 Years After the 2016 Fort McMurray Wildfire: Findings From 9,376 Survey Responses

**DOI:** 10.3389/fpsyt.2021.676256

**Published:** 2021-05-20

**Authors:** Matthew R. G. Brown, Hannah Pazderka, Vincent I. O. Agyapong, Andrew J. Greenshaw, Ivor Cribben, Pamela Brett-MacLean, Julie Drolet, Caroline B. McDonald-Harker, Joy Omeje, Bonnie Lee, Monica Mankowsi, Shannon Noble, Deborah T. Kitching, Peter H. Silverstone

**Affiliations:** ^1^Department of Computing Science, University of Alberta, Edmonton, AB, Canada; ^2^Department of Psychiatry, University of Alberta, Edmonton, AB, Canada; ^3^Department of Accounting and Business Analytics, Alberta School of Business, University of Alberta, Edmonton, AB, Canada; ^4^Faculty of Social Work, University of Calgary, Calgary, AB, Canada; ^5^Department of Sociology and Anthropology, Mount Royal University, Calgary, AB, Canada; ^6^Faculty of Health Sciences, University of Lethbridge, Lethbridge, AB, Canada; ^7^Fort McMurray Catholic School District, Fort McMurray, AB, Canada; ^8^Fort McMurray Public School District, Fort McMurray, AB, Canada

**Keywords:** youth, population mental health, wildfire natural disaster, post-traumatic stress disorder (PTSD), depression, anxiety, alcohol and substance use, resilience

## Abstract

In Fort McMurray, Alberta, Canada, the wildfire of May 2016 forced the population of 88,000 to rapidly evacuate in a traumatic and chaotic manner. Ten percentage of the homes in the city were destroyed, and many more structures were damaged. Since youth are particularly vulnerable to negative effects of natural disasters, we examined possible long-term psychological impacts. To assess this, we partnered with Fort McMurray Public and Catholic Schools, who surveyed Grade 7–12 students (aged 11–19) in November 2017, 2018, and 2019—i.e., at 1.5, 2.5, and 3.5 years after the wildfire. The survey included validated measurement scales for post-traumatic stress disorder (PTSD), depression, anxiety, drug use, alcohol use, tobacco use, quality of life, self-esteem, and resilience. Data analysis was done on large-scale anonymous surveys including 3,070 samples in 2017; 3,265 samples in 2018; and 3,041 samples in 2019. The results were unexpected and showed that all mental health symptoms increased from 2017 to 2019, with the exception of tobacco use. Consistent with this pattern, self-esteem and quality of life scores decreased. Resilience scores did not change significantly. Thus, mental health measures worsened, in contrast to our initial hypothesis that they would improve over time. Of note, we observed higher levels of mental health distress among older students, in females compared to male students, and in individuals with a minority gender identity, including transgender and gender-non-conforming individuals. These findings demonstrate that deleterious mental health effects can persist in youth for years following a wildfire disaster. This highlights the need for multi-year mental health support programs for youth in post-disaster situations. The indication that multi-year, post-disaster support is warranted is relatively novel, although not unknown. There is a need to systematically investigate factors associated with youth recovery following a wildfire disaster, as well as efficacy of psychosocial strategies during later phases of disaster recovery relative to early post-disaster interventions.

## Introduction

In May 2016, a large wildfire affected Fort McMurray, Alberta, Canada and the surrounding area. Called “The Beast” in the popular media (1), the wildfire necessitated the evacuation of the entire city on May 3, 2016, and over 88,000 residents were displaced for several weeks. In addition to damaging community structures and infrastructure, the fire destroyed 10% of the homes in Fort McMurray, leaving many individuals homeless. The fire burned 590,000 hectares of land before it was brought under control on July 4, 2016. Insurance costs for the damages were estimated at $3.6 billion by the Insurance Bureau of Canada, which made the Fort McMurray wildfire the most expensive insured catastrophe in Canadian history (2). Many individuals were left jobless due to damage and closure of local businesses. Social, emotional, and psychological difficulties also affected the community, as is typical after severe disasters (3, 4).

Disasters tend to impact children and youth in particular (5–10) given developmental vulnerabilities (cognitive, emotional, social, and physiological) associated with childhood and adolescence, including the need to rely on others for support (11). Our group has previously examined Fort McMurray school mental health surveys completed by Grade 7–12 students in November 2017 (12, 13). Brown et al. (12) reported elevated mental health symptoms for PTSD and depression compared to a control population from the same province which had not experienced a natural disaster. As reported in (13), individuals who were more personally impacted by the 2016 wildfire, such as having their home destroyed, exhibited greater symptoms of PTSD, depression, anxiety, and alcohol and/or substance misuse. These findings were consistent with previous findings of altered mental health in youth following natural disasters (9, 14).

Previous studies have also reported links between wildfires and adverse mental health effects in adults and children, including increased symptoms of PTSD (15–24), increased symptoms of depression and anxiety (17, 19, 23–28), mental health difficulties (18, 29, 30), reduction in health and well-being (31), increased consumption of anxiolytics-hypnotics (32), and decreased capacity to cope with adversity (33). The combined adverse impacts of wildfire disasters on individuals' mental health and coping abilities, particularly in youth, are important because coping plays a major role in post-disaster recovery, and lack thereof can be detrimental to long-term mental health outcomes [also see (34)]. More generally, non-wildfire disasters, including floods, earthquakes, and tsunamis, have also been linked to adverse effects on mental health in adults and children in the form of increased symptoms or incidences of PTSD, major depressive disorder, suicidality, generalised anxiety disorder, and substance use disorder [see (8, 9, 35–39)]. Displacement caused by natural disasters (e.g., short- and long-term evacuation, homelessness) imposes additional physical, emotional, and psychosocial stress on families, which may then adversely affect longer-term development of children and youth (40).

Longitudinal studies, most of which have focused on adults, have reported long-term negative impacts on mental health from natural disasters (17, 25, 41–46). The pattern of long-term effects can be complex, with individuals exhibiting better recovery from some symptoms, such as depression or anxiety, but lesser recovery for others such as PTSD symptoms (47). Relatively few longitudinal studies have focused on mental health outcomes among youth following natural disaster, although studies of children and youth following Hurricane Katrina have shown long term impacts on mental health (48), with varying recovery trajectories for different groups (49, 50). We are aware of only three population studies that examined wildfire impacts on youth mental health, including one study focusing on PTSD and depression (19) and our previous Fort McMurray studies (12, 13).

In the present study, our hypothesis was that mental health symptoms among Fort McMurray youth would improve with time following the November 2017 survey. This hypothesis was based on theoretical work on trauma recovery, which consistently emphasises the role of time in the recovery process (4, 51, 52). To test this hypothesis, we examined mental health survey data collected by Fort McMurray school boards in Grades 7–12 in November across three consecutive years, including 2017, 2018, and 2019. This repeated testing was conducted 1.5, 2.5, and 3.5 years after the 2016 wildfire (All data were collected prior to the COVID-19 pandemic). We hypothesised specifically that symptoms of PTSD, depression, anxiety, and alcohol/substance use would steadily improve from 2017 to 2018 to 2019.

## Materials and Methods

### Overview and Ethical Considerations

Information collected from students included questions on demographics, mental health, resilience, and personal exposure to and direct impacts of the wildfire. Measurement instruments were selected by the school systems, informed by the relevant literature and advice from the University of Alberta research team. Written letters were sent to parents and guardians to inform them of the survey 2 weeks prior. Parents and guardians could have their child(ren) not participate in the survey, and students also independently had the option to participate or not in the survey as explained at the start of each survey session (see details below and Appendix A: Survey Description Script in the Supplementary Material). Survey data collection was intentionally anonymous, and participants were not asked for their names nor any other identifying information. The study design was approved by the University of Alberta's Health Research Ethics Board (ethics protocol number Pro00072669 approved June 26, 2017). This paper reports on findings from the anonymous survey data collected from both school boards.

Mental health surveys were conducted by the two school boards in Fort McMurray—Fort McMurray Public Schools and Fort McMurray Catholic Schools (henceforth, “Schools”). They asked all students in Grades 7–12 to complete the surveys in November 2017 (18 months after the 2016 wildfire), November 2018 (30 months post-wildfire), and November 2019 (42 months post-wildfire) (All data were collected before the COVID-19 pandemic). The survey was administered during regular class time as part of the standard curriculum to evaluate the support programs the Schools had put in place following the wildfire (see Appendix B: Mental Health Support Programs, in the Supplementary Material). The Schools determined that surveys would be done in November 2017, 2018, and 2019 as the month of November worked best given various logistical and staff capacity considerations.

Fort McMurray Public Schools and Fort McMurray Catholic Schools (“Schools”) administered all aspects of survey data collection, including participant consent, in accordance with their standard procedures and policies. The Schools asked researchers from the University of Alberta for assistance in designing the survey and analysing the anonymous dataset.

### Survey Questionnaires

The survey included 10 questionnaires (see Table 1 for additional details):

1. Demographics Questionnaire (Demographics, 7 questions)—a custom-designed questionnaire assessing age, gender, and the student's grade and school.
2. Impact of Fire Questionnaire (IOF, 6 questions)—a custom-designed questionnaire to assess the impact of the 2016 wildfire on the student.
3. Child PTSD Symptom Scale (CPSS, 19 questions)—used to assess symptoms of post-traumatic stress disorder (PTSD) (53); total CPSS score ranges from 0 to 51.
4. Patient Health Questionnaire, Adolescent version (PHQ-A, 11 questions)—used to assess symptoms of depression and suicidality (54, 55); total PHQ-A score ranges from 0 to 27.
5. Hospital Anxiety and Depression Scale (HADS, 7 questions, anxiety-related questions only)—used to assess symptoms of anxiety (56); total HADS score ranges from 0 to 21.
6. CRAFFT Questionnaire (CRAFFT, 9 questions)—used to assess symptoms of alcohol and substance misuse (57, 58); total CRAFFT scores ranges from 0 to 6.
7. Tobacco Use Questionnaire (2 questions) – two questions on tobacco use: “During the past month, did you smoke tobacco products?” and “During the past month, did you use smokeless tobacco products?”
8. Rosenberg Self-Esteem Scale (Rosenberg, 10 questions)—used to assess self-esteem (59); total score ranges from 0 to 30 with higher scores indicating higher self-esteem.
9. Kidscreen Questionnaire (Kidscreen-10, 11 questions)—used to assess quality of life (60); total Kidscreen-10 score ranges from 0 to 44 with higher scores indicating better quality of life.
10. Child and Youth Resilience Measure (CYRM-12, 12 questions)—used to assess resilience to adverse experience or trauma (61); total CYRM-12 score ranges from 12 to 60.

**Table 1:** Questionnaire details [table reproduced with permission from our previously-published paper Brown et al. (13)].

	**Questions**	**Answer choices**
**Demographics questionnaire**		
1	Are you at school right now, while you are taking the survey?	Yes, No
2	Are you a student?	Yes, No
3	What gender do you identify with?	Female, Male, Other, Prefer not to say
4	What is your age?	10 years or less, 11 years, 12 years, 13 years, 14 years, 15 years, 16 years, 17 years, 18 years, 19 years, 20 years or more
5	What school are you in currently?	Select from a list of all Ft McMurray schools with any classes in grades 7–12
6	What grade are you in currently?	7, 8, 9, 10, 11, 12, other
7	What school were you in for grade 6?	Select from a list of all Ft McMurray schools with grade 6
**Impact of fire questionnaire**		
1	Were in you or near Fort McMurray during any part of the 2016 wildfire?	Yes, No
2	Did you evacuate because of the fire?	Yes, No
3	Was your home destroyed by the fire?	Yes, No
4	Did you see the fire in person?	Yes, No
5	What school are you in?	Select from a list of all Ft McMurray schools with any classes in grades 7–12
6	What grade are you in?	7, 8, 9, 10, 11, 12, other
**PATIENT HEALTH QUESTIONNAIRE (PHQ-A, DEPRESSION SYMPTOMS)**		
**Over the past 2 weeks, how often have you been bothered by any of the following problems?**		
1	Feeling down, depressed, irritable or hopeless	Not at all, Several days, More than half the days, Nearly every day
2	Little interest or pleasure in doing things?	Not at all, Several days, More than half the days, Nearly every day
3	Trouble falling or staying asleep, or sleeping too much	Not at all, Several days, More than half the days, Nearly every day
4	Poor appetite, weight loss, or overeating?	Not at all, Several days, More than half the days, Nearly every day
5	Feeling tired, or having little energy?	Not at all, Several days, More than half the days, Nearly every day
6	Feeling bad about yourself-or that you are a failure or that you have let yourself or your family down	Not at all, Several days, More than half the days, Nearly every day
7	Trouble concentrating on things, such as school work, reading or watching television	Not at all, Several days, More than half the days, Nearly every day
8	Moving or speaking so slowly that other people could have noticed. Or the opposite-being so figety or restless that you have been moving around a lot more than usual	Not at all, Several days, More than half the days, Nearly every day
9	Thoughts that you would be better off dead, or of hurting yourself in some way	Not at all, Several days, More than half the days, Nearly every day
**Questions 10 and 11 asked only if answer to question 9 is not “Not at all”**		
10	Has there been a time in the past month when you have had serious thoughts about ending your life?	Yes, No
11	Have you ever, in your WHOLE LIFE, tried to kill yourself or made a suicide attempt?	Yes, No
**HOSPITAL ANXIETY AND DEPRESSION SCALE (HADS, ANXIETY SYMPTOMS)**		
**Tick the box beside the reply that is closest to how you have been feeling in the past week. Don't take too long over you replies: your immediate is best**		
1	I feel tense or wound up:	Most of the time; A lot of the time; From time to time, occasionally; Not at all
2	I get a sort of frightened feeling as if something bad is about to happen:	Very definitely and quite badly; Yes, but not too badly; A little, but it doesn't worry me; Not at all
3	Worrying thoughts go through my mind:	A great deal of the time; A lot of the time; From time to time, but not too often; Only occasionally
4	I can sit at ease and feel relaxed:	Definitely, Usually, Not often, Not at all
5	I get a sort of frightened feeling like 'butterflies' in the stomach:	Not at all, Occasionally, Quite often, Very often
6	I feel restless and have to be on the move:	Very much indeed, Quite a lot, Not very much, Not at all
7	I get sudden feelings of panic:	Very often indeed, Quite often, Not very often, Not at all
**CPSS QUESTIONNAIRE (PTSD SYMPTOMS)**		
**Instructions to participant: below is a list of problems that kids sometimes have after experiencing an upsetting event. Read each one carefully**		
**and circle the number (0–3) that best describes how often that problem has bothered you in the last 2 weeks**		
1	Please select your most distressing event:	2016 Fort McMurray wildfire, Death of someone close to you, Injury that you suffered, Physical assault against you, Sexual assualt, Other
2	How long as it been since the event (in years)?	<1 month, 2–5 months, 6–11 months, 1 year, 2 years, 3–5 years, 6–10 years, 11 or more years
**Below is a list of problems that kids sometimes have after experiencing an upsetting event. Read each one carefully and circle the number (0–3)**		
**that best describes how often that problem has bothered you in the last 2 weeks**		
3	Having upsetting thoughts or images about the event that came into your head when you didn't want them to	Not at all or only at one time, Once a week or less/once in a while, 2 to 4 times a week/half the time, 5 or more times a week/almost always
4	Having bad dreams or nightmares	Same as above
5	Acting or feeling as if the event was happening again (hearing something or seeing a picture about it and feeling as if I am there again)	Same as above
6	Feeling upset when you think about it or hear about the event (for e.g., feeling scared, angry, sad, guilty, etc)	Same as above
7	Having feelings in your body when you think about or hear about the event (for e.g., breaking out into a sweat, heart beating fast)	Same as above
8	Trying not to think about, talk about, or have feelings about the event	Same as above
9	Trying to avoid activities, people, or places that remind you of the traumatic event	Same as above
10	Not being able to remember an important part of the upsetting event	Same as above
11	Having much less interest or doing things you used to do	Same as above
12	Not feeling close to people around you	Same as above
13	Not being able to have strong feelings (for e.g., being unable to cry or unable to feel happy)	Same as above
14	Feeling as if your future plans or hopes will not come true (for e.g., you will not have a job or getting married or having kids)	Same as above
15	Having trouble falling or staying asleep	Same as above
16	Feeling irritable or having fits of anger	Same as above
17	Having trouble concentrating (for e.g., losing track of a storey on the television, forgetting what you read, not paying attention in class)	Same as above
18	Being overly careful (for e.g., checking to see who is around you and what is around you)	Same as above
19	Being jumpy or easily startled (for e.g., when someone walks up behind you)	Same as above
**CRAFFT QUESTIONNAIRE (DRUGS/ALCOHOL/TABACCO)**		
**During the past 12 months, did you:**		
1	Drink any alcohol (more than a few sips)?	Yes, No
2	Smoke any marijuana or hashish?	Yes, No
3	Use anything else to get high?	Yes, No
4	Have you ever ridden in a CAR driven by someone (including yourself) who was “high” or had been using alcohol or drugs?	Yes, No
**Questions 5–9 asked only if “yes” to one or more of questions 1–3**		
5	Do you ever use alcohol or drugs to RELAX, feel better about yourself, or fit in?	Yes, No
6	Do you ever use alcohol or drugs while you are by yourself, or ALONE?	Yes, No
7	Do you every FORGET things you did while using alcohol or drugs?	Yes, No
8	Do your FAMILY or FRIENDS ever tell you that you should cut down on your drinking or drug use?	Yes, No
9	Have you ever gotten into TROUBLE while you were using alcohol or drugs?	Yes, No
**Tobacco use questionnaire**		
1	During the past month, did you smoke tobacco products?	Yes, No
2	During the past month, did you use smokeless tobacco products?	Yes, No
**Rosenberg self-esteem scale**		
1	On the whole, I am satisfied with myself	Strongly agree, Agree, Disagree, Strongly disagree
2	At times, I think I am no good at all	Strongly agree, Agree, Disagree, Strongly disagree
3	I feel that I have a number of good qualities	Strongly agree, Agree, Disagree, Strongly disagree
4	I am able to do things as well as most other people	Strongly agree, Agree, Disagree, Strongly disagree
5	I feel I do not have much to be proud of	Strongly agree, Agree, Disagree, Strongly disagree
6	I certainly feel useless at times	Strongly agree, Agree, Disagree, Strongly disagree
7	I feel that I'm a person of worth, at least on an equal plane with others	Strongly agree, Agree, Disagree, Strongly disagree
8	I wish I could have more respect for myself	Strongly agree, Agree, Disagree, Strongly disagree
9	All in all, I am inclined to feel that I am a failure	Strongly agree, Agree, Disagree, Strongly disagree
10	I take a positive attitude towards myself	Strongly agree, Agree, Disagree, Strongly disagree
**KIDSCREEN QUESTIONNAIRE (QUALITY OF LIFE)**		
**Thinking about the last week:**		
1	Have you physically felt fit and well?	Not at all, Slightly, Moderately, Very, Extremely
2	Have you felt full of energy?	Never, Seldom, Quite often, Very often, Always
3	Have you felt sad?	Never, Seldom, Quite often, Very often, Always
4	Have you felt lonely?	Never, Seldom, Quite often, Very often, Always
5	Have you had enough time for yourself?	Never, Seldom, Quite often, Very often, Always
6	Have you been able to do the things that you want to do in your free time?	Never, Seldom, Quite often, Very often, Always
7	Have your parent(s) treated you fairly?	Never, Seldom, Quite often, Very often, Always
8	Have you had fun with your friends?	Never, Seldom, Quite often, Very often, Always
9	Have you got on well at school?	Not at all, Slightly, Moderately, Very, Extremely
10	Have you been able to pay attention?	Never, Seldom, Quite often, Very often, Always
11	In general, how would you say your health is?	Excellent, Very good, Good, Fair, Poor
**CHILD AND YOUTH RESILIENCE MEASURE (CYRM-12)**		
**To what extent do the sentences below describe you? Select an answer for each statement**		
1	I am able to solve my problems without harming myself or others	Not at all, A little, Some-what, Quite a bit, A lot
2	I know where to go in the community to get help	Not at all, A little, Some-what, Quite a bit, A lot
3	Getting an education is important to me	Not at all, A little, Some-what, Quite a bit, A lot
4	I try to finish what I start	Not at all, A little, Some-what, Quite a bit, A lot
5	I have people I look up to	Not at all, A little, Some-what, Quite a bit, A lot
6	My parents/caregivers know a lot about me	Not at all, A little, Some-what, Quite a bit, A lot
7	My family stands by me during difficult times	Not at all, A little, Some-what, Quite a bit, A lot
8	My friends stand by me during difficult times	Not at all, A little, Some-what, Quite a bit, A lot
9	I have opportunities to develop skills that will be useful later in life	Not at all, A little, Some-what, Quite a bit, A lot
10	I am treated fairly in my community	Not at all, A little, Some-what, Quite a bit, A lot
11	I feel I belong at school	Not at all, A little, Some-what, Quite a bit, A lot
12	I enjoy my cultural and family traditions	Not at all, A little, Some-what, Quite a bit, A lot

### Survey Administration Procedure

The vast majority (>98%) of students who completed the survey did so during regular school hours. A few students citing special circumstances completed the survey from home on their own computers. Depending on their school, students either completed the survey using a desktop computer in a computer laboratory or used laptops brought to their classroom. The survey website was based on an HTML/CSS front end and a back end server written in the Clojure programming language (http://clojure.org). A survey description script was read to each class at the beginning of the survey session (reproduced in the Supplementary Material). The script explained the purpose of the survey and provided instructions for completing the survey. It also explained that the survey was anonymous (participants were not asked for their names nor date of birth) and that participation was voluntary. Students had an opportunity to ask questions before participating. The survey battery included 96 questions in total. Participation required <20 min for most students, but a small number of students took up to 50 min. Participants were able to skip questions, but the survey description script and the survey website did encourage them to answer all questions.

### Repeated Measurements in 2017, 2018, and 2019

The first mental health survey of Fort McMurray Grade 7–12 students was completed in November 2017 and was then repeated in November 2018 and November 2019. Many students were present for the survey across all 3 years. Other students were present for only 1 or 2 years. For example, students entering Grade 7 in September 2018 or September 2019 were not surveyed in *earlier* years (i.e., November 2017 and November 2018, respectively). Similarly, students graduating from Grade 12 in June 2018 or June 2019 were not re-surveyed the following November. Some students also moved to, or away from, Fort McMurray between 2017 and 2019. In summary, some of the surveys collected in 2017, 2018, and 2019 were completed by unique students (i.e., no repetition), although the majority were completed by the same students (i.e., repeated measures over 2 or 3 years). Because survey responses were anonymous, we were unable to link surveys completed by specific students from 1 year to the next. We therefore employed a between-subject analysis (treating all survey samples as independent) rather than using a repeated-measures, within-subject analysis over successive years. It is important to note that adopting a between-subject approach is statistically conservative as there is an expectation of increased overall error variance compared to the likely advantage of within-subject analysis.

### Cut-Off Scores and Probable Diagnoses

Probable diagnoses of four different psychiatric conditions were established by thresholding each participant's scores on specific scales. Threshold values for probable diagnoses were derived from the relevant literature for each scale, as described below. Specific probable diagnoses included PTSD (based on the CPSS scale), depression (from the PHQ-A), anxiety (from the HADS), and alcohol/substance use disorder (based on the CRAFFT).

The term “probable diagnosis” is used here, as opposed to “clinical diagnosis,” because the scores were based on self-report scales rather than psychiatric clinical interviews. The literature reports good agreement between psychiatric clinical diagnoses of PTSD, depression, anxiety, and alcohol/substance use disorder with probable diagnoses derived from widely-published threshold scores for the above four questionnaires (57, 58, 62–65), and we have previously used this approach (66).

Probable PTSD was determined based on a CPSS score of 15 or more (65, 67). Probable depression was determined based on a PHQ-A score of 11 or more (63). Probable moderately severe depression was determined based on a PHQ-A score of 15 or more (62). Suicidal thinking was determined from responses to two questions from the PHQ-A: question 9 “Over the past 2 weeks, how often have you been bothered by any of the following problems: Thoughts that you would be better off dead, or of hurting yourself in some way?” and question 10 “Has there been a time in the past month when you have had serious thoughts about ending your life?” Participants were assessed as exhibiting suicidal thinking if they answered “Several days,” “More than half the days,” or “Nearly every day” to PHQ-A question 9 and “Yes” to question 10. Participants answering “Not at all” to question 9 skipped (were not shown) question 10, and they were assessed as not exhibiting suicidal thinking. In addition, participants answering “Several days,” “More than half the days,” or “Nearly every day” to PHQ-A question 9 and “No” to question 10 were assessed as not exhibiting suicidal thinking (as distinct from thinking about self-harm) (PHQ-A question 11 was not considered in the definition of suicidal thinking). Probable anxiety was determined based on a HADS score of 11 or more (64). Probable alcohol/substance use disorder was determined based on a CRAFFT score of 2 or more (57, 58). Tobacco use was determined as answering “yes” to either of the two questions on the Tobacco Use Questionnaire. Finally, an “Any of 4 probable diagnoses” criterion was defined as being positive for one or more of the four probable diagnoses: PTSD, depression, anxiety, or alcohol/substance use disorder.

### Dependent Variables and Statistical Effects Tested

We examined the following 15 dependent measures: (1) CPSS PTSD score, (2) PHQ-A depression score, (3) HADS anxiety score, (4) CRAFFT alcohol/substance use score, (5) Rosenberg self-esteem score, (6) Kidscreen quality of life score, (7) CYRM-12 resilience score, (8) percent probable PTSD, (9) percent probable depression, (10) percent probable moderately severe depression, (11) percent suicidal thinking, (12) percent probable anxiety, (13) percent probable alcohol/substance use disorder, (14) percent tobacco use, and (15) percent any of four probable diagnoses (See “Cut-off Scores and Probable Diagnoses” section above for details of scoring cut-offs used to determine probable diagnoses). In analysing data for a given measure (e.g., mean CPSS) or probable diagnosis (e.g., probable PTSD), we included only those participants who provided answers for all questions in the relevant questionnaire.

For each of the 15 dependent measures, we tested five statistical effects: (1) linear effect of time (2017 vs. 2018 vs. 2019), (2) linear effect of age (11 to 19 years old), (3) effect of female vs. male gender identity, (4) effect of other vs. female/male gender identity, and (5) effect of preferred not to say vs. female/male gender identity.

Participant age was determined by Demographics Questionnaire question 4 “What is your age?,” with answer choices “10 years or less,” “11 years,” “12 years,” “13 years,” “14 years,” “15 years,” “16 years,” “17 years,” “18 years,” “19 years,” and “20 years or more.” Note that participants answering “10 years or less” or “20 years or more” were excluded to enable modelling of age as a linear variable (see “Data Exclusion” section in the Results).

Participant gender identity was determined based on Demographics Questionnaire question 3 “What gender do you identify with?,” with answer choices “female,” “male,” “other,” and “prefer not to say.” Statistical test 3 (female vs. male) compared participants answering “female” vs. those answering “male.” Test 4 (other vs. female/male) compared participants answering “other” vs. those answering either “female” or “male.” Test 5 (preferred not to say vs. female/male) compared participants answering “prefer not to say” vs. those answering either “female” or “male.” Each test of a gender effect included only those participants with the relevant gender identities (test 3: female and male; test 4: female, male, and other; test 5: female, male, and preferred not to say).

### Details of Statistical Analysis

All statistical comparisons were done using permutation testing on the slope parameter from a fitted linear model, with a null hypothesis of zero slope. The linear model included a slope parameter for the effect variable (time, age, female vs. male, other vs. female/male, preferred not to say vs. female/male) as well as parameters for “nuisance variables” as described below. Mathematical details of the linear modelling procedure are included in the “Details of Linear Modelling” section below.

Permutation testing is a non-parametric method and was chosen for its robustness against non-normality. The number of iterations was 10^5^ for all permutation tests. All tests were two-tailed (That is, to compute the *p*-value for a given test, the absolute value of the slope parameter fitted to the real data was compared against the absolute values of the 10^5^ simulated slope parameters fitted to permuted data).

In total, our analysis of the five statistical effects for each of 15 dependent variables included 75 individual statistical tests. We addressed multiple comparisons using the Benjamini-Hochberg method for false discovery rate (FDR) correction. This method computed a threshold of *p* = 0.025 for FDR correction across all 75 tests.

Distributions of gender identities and ages were similar across time, and the distribution of gender identities was similar across different ages (see “Demographics” in the Results section). Nonetheless, to address the possibility that results for one effect might have been driven in part by some small difference in one or more of the other effect variables, we included “nuisance variables” in the linear models to which permutation testing was applied. To test effects of time (2017 vs. 2018 vs. 2019), we used a linear model with a term for time as well as nuisance variables including a covariate for age and four indicator variables for gender identity: female, male, other, and preferred not to say. For analyses on the effects of time, only the fitted slope parameter for time was used to generate *p*-values. Including the other nuisance variables allowed the model to separate out effects of age and gender from effects of time. Similarly, analyses of age used five nuisance variables, including a time covariate and four indicator variables for gender identities. Analyses of gender effects (female vs. male, other vs. female/male, preferred not to say vs. female/male) included time and age as nuisance variables.

As expected from a sample of students in Grade 7–12, there were substantially fewer participants who were 11 or 18–19 years old, compared to those who were 12–17 years old, at the time of data collection (see “Demographics” in the Results section). To address the possibility that results for effects of age might be driven by leverage effects from smaller sample numbers in the extremes (aged 11 or 18–19 years old), we ran a follow-up analysis which included only participants aged 12–17.

We performed all analyses using in-house computer code written in the Clojure programming language (http://clojure.org). The code for statistical testing and FDR correction is available at http://github.com/mbrown/mrgbstats.

### Details of Linear Modelling

For a given analysis, we defined an effect variable *x*_*i*_ and a dependent variable *y*_*i*_, as well as *J* covariate nuisance variables *v*_*j, i*_ and *K* indicator nuisance variables *w*_*k, i*_, where *i*∈[1, *N*] with *N* being the number of surveys used in the analysis.

For analyses on effects of time or effects of age, the effect variable *x*_*i*_ was mean-centred to make it “independent” (orthogonal) to the intercept (i.e., constant offset). For analyses on effects of gender (female vs. male, other vs. female/male, preferred not to say vs. female/male), the effect variable was categorical and therefore not mean-centred. Covariate nuisance variables *v*_*j, i*_ were mean-centred, and indicator nuisance variables *w*_*k, i*_ were not mean-centred.

For analyses on effects of time or effects of age, we did not include an intercept term in the model (i.e., a constant offset column containing all ones in the model matrix). Because analyses of time and age included four nuisance indicator variables for gender identities, an intercept column would have been a linear combination of those four indicator variables, rendering the model matrix degenerate. The effect variable for time or for age was mean-centred and therefore orthogonal to the constant offset, in any case. For analyses of effects of gender, which did not have the four nuisance variables for gender, we did include a constant offset term.

For each analysis, we created a model matrix ***X*** from the effect variable *x*_*i*_, *J* covariate nuisance variables *v*_*j, i*_, and *K* indicator nuisance variables *w*_*k, i*_, as well as a constant offset term for analyses of gender effects. Each survey *i* contributed one effect variable value, one dependent variable value, and one value for each of (*J* + *K*) nuisance variables. For example, for analyses on effects of time, *x*_*i*_ was time; *v*_1, *i*_ was age; *w*_1, *i*_, *w*_2, *i*_, *w*_3, *i*_, and *w*_4, *i*_ were indicator variables for gender identity, including female, male, other, and preferred not to say; and there was no constant offset column. To take a second example, for analyses of other vs. female/male gender identity, *x*_*i*_ was 0 for participants identifying as female or male and 1 for those with other gender identity; *v*_1, *i*_ and *v*_2, *i*_ were time and age, respectively; there were no indicator nuisance variables; and there was a constant offset column. We created a model matrix ***X*** with size *N by* (1 + *J* + *K*) or else *N by* (2 + *J* + *K*), for analyses without and with a constant offset column, respectively. The first column of ***X*** consisted of the effect variable values *x*_*i*_. If a constant offset column was included, it was the second column. The next *J* columns were comprised of the mean-centred covariate nuisance variables *v*_*j, i*_, and the last *K* columns were comprised of the indicator nuisance variables *w*_*k, i*_. We defined the vector $\hat{\beta }={{(X}^{T}X)}^{-1}X^{T}\overset{⇀}{y}$ where $\overset{⇀}{y}$was the vector of dependent variable values *y*_*i*_. The slope parameter used for permutation testing was ${\hat{\beta }}_{1}$, the first element of $\hat{\beta }$, representing the fitted scaling parameter for the effect variable.

## Results

The survey was administered to all Grade 7–12 students in Fort McMurray, Alberta, Canada who were attending either the Public or Catholic schools on the days the survey was conducted during the month of November in 2017, 2018, and 2019. Five Public schools and two Catholic schools participated in the survey. In total, 9,920 surveys were collected during the period of 2017 to 2019. Forty five percentage were collected from Public schools and 55% from Catholic schools. As all surveys were anonymous, there was no way to identify which students did or did not repeat the survey over successive years.

### Data Exclusion

Data from 544 surveys were excluded based the following exclusion criteria:

1. Participant answered “10 years or less” to the age question (Demographics Questionnaire question 4).
2. Participant answered “20 years or more” to the age question (Demographics Questionnaire question 4).
3. Participant gave inconsistent answers for positive and negative questions included in the Rosenberg questionnaire (for details, see Appendix C: Exclusion Criteria in the Supplementary Material).
4. Participant gave inconsistent answers for positive questions from the Rosenberg questionnaire and positive questions from the Kidscreen questionnaire (see Appendix C: Exclusion Criteria the Supplementary Material, for details).
5. Participant gave inconsistent answers among the non-reversed and reversed questions from the HADS questionnaire (For two of the HADS questions, the answer order is reversed to test for consistency; see details in Supplementary Material).

Criteria 1 and 2 above excluded participants with ambiguous age. The remaining participants had non-ambiguous ages in the range 11–19 years, allowing us to model effects of age as a linear variable. Criteria 3 to 5 above excluded participants who gave inconsistent answers, possibly because they were not paying attention to the survey or did not understand the questions. After exclusions, the final dataset included 9,376 surveys.

### Demographics

Demographics for the 9,376 surveys were as follows. Self-reported gender identity was 47.3% female, 48.6% male, 1.7% other, and 2.4% preferred not to say. Age ranged from 11 to 19, and the mean age of participants was 14.3 years ± 1.8 (standard deviation). For additional demographic details, see Table 2. Distributions of gender identities and ages were similar across the 3 years of data collection (Table 2). The distribution of gender identities was similar across different ages as well (Table 2).

**Table 2:** Demographics.

	**Total collected**		**After exclusions**		**Total students enrolled**			**Recruitment rate**	
**Number surveys collected and recruitment rates**									
All 3 years	9,920		9,376		13,848			67.7%	
2017	3,252		3,070		4,407			69.7%	
2018	3,451		3,265		4,592			71.1%	
2019	3,217		3,041		4,849			62.7%	
	**Female**		**Male**		**Other**			**Did not say**	
**Gender distributions**									
All 3 years	47.3%		48.6%		1.7%			2.4%	
2017	48.3%		47.8%		1.6%			2.4%	
2018	46.6%		49.9%		1.6%			2.0%	
2019	47.0%		48.0%		1.9%			3.0%	
	**11 yrs**	**12 yrs**	**13 yrs**	**14 yrs**	**15 yrs**	**16 yrs**	**17 yrs**	**18 yrs**	**19 yrs**
**Age distributions**									
All 3 years	2.7%	18.1%	17.7%	17.2%	16.4%	14.5%	11.0%	1.9%	0.6%
2017	2.4%	17.9%	17.2%	17.7%	15.5%	14.4%	12.2%	2.0%	0.7%
2018	2.6%	17.7%	17.2%	16.3%	17.9%	15.4%	10.2%	2.2%	0.6%
2019	3.0%	18.7%	18.8%	17.7%	15.7%	13.7%	10.6%	1.5%	0.4%
	**11 yrs**	**12 yrs**	**13 yrs**	**14 yrs**	**15 yrs**	**16 yrs**	**17 yrs**	**18 yrs**	**19 yrs**
**Age distributions by gender**									
All	2.7%	18.1%	17.7%	17.2%	16.4%	14.5%	11.0%	1.9%	0.6%
Female	2.9%	17.9%	17.2%	17.4%	16.7%	14.4%	11.2%	1.7%	0.6%
Male	2.3%	18.5%	17.7%	17.2%	16.2%	14.6%	10.8%	2.1%	0.5%
Other	4.5%	7.1%	23.7%	16.7%	18.6%	14.7%	11.5%	3.2%	0.0%
Did Not Say	3.1%	19.6%	24.0%	16.0%	13.3%	13.3%	8.9%	0.9%	0.9%

“*Did Not Say” refers to participants who answered the question on gender identity with “Prefer not to say.”*

### Changes Over Time (2017, 2018, 2019)

Table 3 shows results for analysis of changes over time. Permutation testing of the linear effect of time, with age and gender partialled out, indicated that 13 of the 15 dependent measures changed over time during the period from 2017 to 2019, with statistical significance surviving FDR multiple comparison correction. Scores for PTSD (CPSS), depression (PHQ-A), anxiety (HADS), and alcohol/substance use (CRAFFT) increased from 2017 to 2019. Self-esteem (Rosenberg) and quality of life (Kidscreen) scores decreased. Resilience (CYRM-12) scores did not change significantly. Rates of probable diagnoses of PTSD, depression, moderately severe depression, suicidal thinking, anxiety, alcohol/substance use disorder, and the “Any of 4 probable diagnoses” category all increased. Rates of tobacco use did not change significantly.

**Table 3:** Changes over time (2017, 2018, 2019).

**Measure**	***N* 2017**	***N* 2018**	***N* 2019**	**Score 2017**	**Score 2018**	**Score 2019**	**Slope**	***P*-value**	**FDR**
CPSS score (PTSD)	2,869	3,035	2,829	12.83 ± 11.47	13.03 ± 11.33	13.75 ± 11.87	0.47	0.0025	*
PHQ-A score (depression)	2,964	3,149	2,926	8.02 ± 6.47	8.23 ± 6.48	8.69 ± 6.70	0.36	0.00001	*
HADS score (anxiety)	2,983	3,170	2,939	7.74 ± 4.72	7.72 ± 4.68	8.01 ± 4.79	0.14	0.019	*
CRAFFT score (alcohol/drugs)	2,994	3,190	2,947	0.55 ± 1.26	0.63 ± 1.30	0.61 ± 1.32	0.04	0.014	*
Rosenberg score (self-esteem)	2,967	3,138	2,921	18.22 ± 6.57	18.01 ± 6.51	17.58 ± 6.68	−0.33	0.00011	*
Kidscreen score (quality of life)	2,977	3,171	2,926	26.97 ± 8.19	26.75 ± 8.37	26.40 ± 8.45	−0.33	0.0028	*
CYRM-12 score (resilience)	2,929	3,096	2,857	46.48 ± 9.17	46.97 ± 9.12	46.69 ± 9.31	0.12	0.32	
**Measure**	***N*** **2017**	***N*** **2018**	***N*** **2019**	**Rate 2017**	**Rate 2018**	**Rate 2019**	**Slope**	***P*****-value**	**FDR**
Probable PTSD	2,869	3,035	2,829	37%	39%	41%	2.18	0.00070	*
Probable depression	2,964	3,149	2,926	31%	31%	35%	2.20	0.00033	*
Probable moderately severe depression	2,964	3,149	2,926	17%	18%	20%	1.66	0.0011	*
Suicidal thinking	2,990	3,175	2,948	16%	16%	18%	1.15	0.018	*
Probable anxiety	2,983	3,170	2,939	27%	28%	31%	2.16	0.00020	*
Probable alcohol/substance use disorder	2,994	3,190	2,947	15%	17%	16%	1.21	0.012	*
Tobacco use	3,004	3,201	2,956	13%	15%	12%	−0.16	0.71	
Any of 4 probable diagnoses	3,052	3,240	3,023	46%	49%	51%	2.65	0.00004	*

*Table shows participant numbers, summary statistics, and results for analysis of effects of time. N X columns indicate number of participants from year X. Score X columns show mean ± standard deviation (not standard error) for year X. Rate X columns indicate rates of probable diagnosis for year X. Slope is the fitted slope parameter from a linear model with age and gender partialled out (see discussion of nuisance variables in the Materials and Methods). The slope parameter indicates the linear relationship between time and the dependent variable, independent of age and gender. P-values were derived from permutation testing on the slope parameters. Comparisons with an asterisk under the FDR column survived FDR multiple comparison correction. P-threshold for FDR correction was 0.025*.

### Effects of Age (11–19 Years Old)

Table 4 shows results for the analysis of age effects. Permutation testing of the linear effect of age over the range 11–19 years old, with time and gender partialled out, revealed that all 15 dependent measures changed with age, with statistical significance surviving FDR multiple comparison correction. Scores for PTSD (CPSS), depression (PHQ-A), anxiety (HADS), and alcohol/substance use (CRAFFT) increased with age. Self-esteem (Rosenberg), quality of life (Kidscreen), and resilience (CYRM-12) scores decreased with age. Rates of probable diagnoses of PTSD, depression, moderately severe depression, suicidal thinking, anxiety, alcohol/substance use disorder, tobacco use, and the “Any of 4 probable diagnoses” category all increased with age.

**Table 4:** Effects of age (11–19 years old).

**Measure**	***N* 11**	***N* 12**	***N* 13**	***N* 14**	***N* 15**	***N* 16**	***N* 17**	***N* 18**	***N* 19**	**Score 11**	**Score 12**	**Score 13**	**Score 14**	**Score 15**	**Score 16**	**Score 17**	**Score 18**	**Score 19**	**Slope**	***P*-value**	**FDR**
CPSS score (PTSD)	216	1,540	1,525	1,521	1,444	1,294	977	168	48	13.09 ± 11.19	11.97 ± 10.83	12.99 ± 11.41	13.44 ± 11.68	12.61 ± 11.51	13.85 ± 11.73	14.39 ± 12.23	16.20 ± 12.21	16.44 ± 11.83	0.42	0.00001	*
PHQ-A score (depression)	225	1,611	1,592	1,572	1,486	1,326	1,002	175	50	7.23 ± 5.78	6.75 ± 5.86	7.62 ± 6.34	8.38 ± 6.69	8.43 ± 6.51	9.33 ± 6.85	10.03 ± 6.77	10.47 ± 6.47	11.28 ± 6.87	0.60	0.00001	*
HADS score (anxiety)	236	1,632	1,597	1,573	1,498	1,328	1,005	173	50	7.40 ± 4.35	7.13 ± 4.46	7.58 ± 4.65	7.72 ± 4.78	7.82 ± 4.73	8.33 ± 4.83	8.71 ± 4.92	8.91 ± 4.73	8.48 ± 4.27	0.27	0.00001	*
CRAFFT score (alcohol/drugs)	236	1,647	1,617	1,578	1,497	1,327	1,007	172	50	0.04 ± 0.32	0.12 ± 0.60	0.30 ± 0.92	0.49 ± 1.13	0.73 ± 1.41	1.01 ± 1.56	1.26 ± 1.68	1.11 ± 1.65	1.92 ± 2.33	0.22	0.00001	*
Rosenberg score (self-esteem)	233	1,618	1,590	1,562	1,481	1,323	998	171	50	18.63 ± 6.36	19.08 ± 6.35	18.13 ± 6.76	17.57 ± 6.76	17.83 ± 6.55	17.60 ± 6.62	17.17 ± 6.35	16.32 ± 5.87	15.84 ± 5.96	−0.33	0.00001	*
Kidscreen score (quality of life)	237	1,625	1,600	1,572	1,491	1,326	998	175	50	28.93 ± 8.20	29.22 ± 8.09	27.92 ± 8.13	26.72 ± 8.45	26.17 ± 7.94	24.92 ± 8.29	24.14 ± 8.11	23.42 ± 7.56	22.42 ± 8.02	−0.97	0.00001	*
CYRM-12 score (resilience)	225	1,585	1,550	1,537	1,470	1,308	988	171	48	47.48 ± 9.36	47.98 ± 8.87	46.46 ± 9.49	46.13 ± 9.46	46.78 ± 9.08	46.64 ± 8.89	46.32 ± 9.01	44.50 ± 9.76	45.73 ± 10.65	−0.25	0.00002	*
**Measure**	***N*** **11**	***N*** **12**	***N*** **13**	***N*** **14**	***N*** **15**	***N*** **16**	***N*** **17**	***N*** **18**	***N*** **19**	**Rate 11**	**Rate 12**	**Rate 13**	**Rate 14**	**Rate 15**	**Rate 16**	**Rate 17**	**Rate 18**	**Rate 19**	**Slope**	***P*****-value**	**FDR**
Probable PTSD	216	1,540	1,525	1,521	1,444	1,294	977	168	48	38%	34%	38%	40%	36%	41%	43%	53%	48%	1.65	0.00001	*
Probable depression	225	1,611	1,592	1,572	1,486	1,326	1,002	175	50	26%	22%	28%	33%	33%	38%	44%	43%	46%	3.68	0.00001	*
Probable moderately severe depression	225	1,611	1,592	1,572	1,486	1,326	1,002	175	50	12%	11%	16%	20%	17%	23%	27%	26%	34%	2.58	0.00001	*
Suicidal thinking	230	1,638	1,608	1,578	1,496	1,328	1,009	176	50	17%	14%	18%	17%	15%	17%	19%	23%	28%	0.57	0.0088	*
Probable anxiety	236	1,632	1,597	1,573	1,498	1,328	1,005	173	50	26%	22%	27%	28%	28%	33%	36%	40%	28%	2.35	0.00001	*
Probable alcohol/substance use disorder	236	1,647	1,617	1,578	1,497	1,327	1,007	172	50	1%	3%	8%	13%	20%	28%	35%	30%	42%	5.93	0.00001	*
Tobacco use	235	1,648	1,620	1,582	1,506	1,335	1,010	175	50	3%	4%	7%	11%	17%	21%	24%	22%	44%	4.01	0.00001	*
Any of 4 probable diagnoses	247	1,683	1,649	1,604	1,525	1,351	1,023	180	53	39%	37%	43%	49%	49%	57%	65%	63%	62%	4.64	0.00001	*

*Table shows participant numbers, summary statistics, and results of analysis of effects of age 11 to 19 years old. N X columns indicate number of participants from age group X. Score X columns show mean ± standard deviation (not standard error) for age group X. Rate X columns indicate rates of probable diagnosis for age group X. Slope is the fitted slope parameter from a linear model with time and gender partialled out (see discussion of nuisance variables in the Materials and Methods). The slope parameter indicates the linear relationship between age and the dependent variable, independent of time and gender. P-values were derived from permutation testing on the slope parameters. Comparisons with an asterisk under the FDR column survived FDR multiple comparison correction. P-threshold for FDR correction was 0.025*.

We did follow-up analyses of age restricted to participants aged 12–17 years old, with time and gender partialled out. Suicidal thinking did not change significantly over time with participants aged 12–17 years (*p* = 0.11), in contrast to analysis of age 11–19 years, which found a statistically significant increase in the rate of suicidal thinking with age (*p* = 0.0088). The other 14 dependent measures exhibited the same pattern of statistically significant changes in analyses with age 12–17 years (CYRM-12 *p* = 0.00038, CPSS *p* = 0.00002, all other tests *p* = 0.00001) as compared to analyses with age 11–19 years.

### Effects of Gender Identity

Tables 5–7 present results of three analyses of gender identity. Gender identity was determined based on Demographics Questionnaire question 3 “What gender do you identify with?,” with answer choices “female,” “male,” “other,” and “prefer not to say.” Analyses of gender identity compared female vs. male (Table 5), other vs. female/male (Table 6), and preferred not to say vs. female/male (Table 7) (See “Dependent Variables and Statistical Effects Tested” in the Materials and Methods section for additional details). Analyses of gender effects were done using permutation testing, with time and age partialled. All 15 dependent measures showed significant differences for all three gender identity comparisons, with statistical significance surviving FDR multiple comparison correction for all tests. Scores for PTSD (CPSS), depression (PHQ-A), anxiety (HADS), and alcohol/substance use (CRAFFT) were higher in females vs. males, in those with other gender identity vs. females/males, and in those who preferred not to say vs. females/males. Self-esteem (Rosenberg), quality of life (Kidscreen), and resilience (CYRM-12) scores were lower in females, in participants with other gender identity, and in participants who preferred not to say. Rates of probable diagnoses of PTSD, depression, moderately severe depression, suicidal thinking, anxiety, alcohol/ substance use disorder, tobacco use, and the “Any of 4 probable diagnoses” category were higher in females, in those with other gender identity, and in those who preferred not to say.

**Table 5:** Effects female vs. male gender identity.

**Measure**	***N* male**	***N* female**	**Score male**	**Score female**	**Slope**	***P*-value**	**FDR**
CPSS score (PTSD)	4,201	4,190	10.61 ± 10.30	15.39 ± 11.96	4.79	0.00001	*
PHQ-A score (depression)	4,391	4,297	6.76 ± 5.88	9.66 ± 6.75	2.90	0.00001	*
HADS score (anxiety)	4,405	4,325	6.52 ± 4.27	9.01 ± 4.77	2.50	0.00001	*
CRAFFT score (alcohol/drugs)	4,438	4,330	0.52 ± 1.20	0.64 ± 1.33	0.12	0.00002	*
Rosenberg score (self-esteem)	4,375	4,287	19.55 ± 6.15	16.53 ± 6.53	−3.02	0.00001	*
Kidscreen score (quality of life)	4,397	4,314	28.91 ± 7.72	24.72 ± 8.29	−4.20	0.00001	*
CYRM-12 score (resilience)	4,292	4,248	47.25 ± 9.05	46.64 ± 9.05	−0.62	0.0014	*
**Measure**	***N*** **male**	***N*** **female**	**Rate male**	**Rate female**	**Slope**	***P*****-value**	**FDR**
Probable PTSD	4,201	4,190	29%	47%	17.60	0.00001	*
Probable depression	4,391	4,297	23%	40%	17.56	0.00001	*
Probable moderately severe depression	4,391	4,297	12%	24%	12.54	0.00001	*
Suicidal thinking	4,426	4,326	12%	21%	8.48	0.00001	*
Probable anxiety	4,405	4,325	17%	38%	21.03	0.00001	*
Probable alcohol/substance use disorder	4,438	4,330	14%	17%	3.46	0.00001	*
Tobacco use	4,451	4,341	12%	14%	2.05	0.0040	*
Any of 4 probable diagnoses	4,532	4,402	39%	58%	19.02	0.00001	*

*Table shows participant numbers, summary statistics, and results of analysis of effects of female vs. male gender identity. N X columns indicate number of participants identifying as gender X (female or male). Score X columns show mean ± standard deviation (not standard error) for gender X. Rate X columns indicate rates of probable diagnosis for gender X. Slope is the fitted slope parameter from a linear model with time and age partialled out (see discussion of nuisance variables in the Materials and Methods). The slope parameter indicates the linear relationship between the female vs. male effect variable and the dependent variable, independent of time and age. P-values were derived from permutation testing on the slope parameters. Comparisons with an asterisk under the FDR column survived FDR multiple comparison correction. P-threshold for FDR correction was 0.025*.

**Table 6:** Effects of other vs. female/male gender identity.

**Measure**	***N* female/male**	***N* other**	**Score female/male**	**Score other**	**Slope**	***P*-value**	**FDR**
CPSS score (PTSD)	8,391	142	13.00 ± 11.42	19.38 ± 14.02	6.30	0.00001	*
PHQ-A score (depression)	8,688	145	8.20 ± 6.49	12.52 ± 7.67	4.18	0.00001	*
HADS score (anxiety)	8,730	150	7.75 ± 4.69	9.93 ± 5.11	2.13	0.00001	*
CRAFFT score (alcohol/drugs)	8,768	149	0.58 ± 1.27	1.13 ± 1.77	0.52	0.00001	*
Rosenberg score (self-esteem)	8,662	148	18.06 ± 6.52	14.25 ± 8.25	−3.74	0.00001	*
Kidscreen score (quality of life)	8,711	148	26.83 ± 8.28	22.98 ± 9.74	−3.64	0.00001	*
CYRM-12 score (resilience)	8,540	139	46.95 ± 9.05	40.28 ± 11.33	−6.64	0.00001	*
**Measure**	***N*** **female/male**	***N*** **other**	**Rate female/male**	**Rate other**	**Slope**	***P*****-value**	**FDR**
Probable PTSD	8,391	142	38%	56%	17.78	0.00003	*
Probable depression	8,688	145	32%	61%	28.19	0.00001	*
Probable moderately severe depression	8,688	145	18%	42%	23.71	0.00001	*
Suicidal thinking	8,752	150	16%	38%	21.54	0.00001	*
Probable anxiety	8,730	150	28%	43%	15.03	0.00006	*
Probable alcohol/substance use disorder	8,768	149	16%	28%	11.51	0.00024	*
Tobacco use	8,792	152	13%	24%	10.13	0.00027	*
Any of 4 probable diagnoses	8,934	156	48%	69%	20.03	0.00002	*

*Table shows participant numbers, summary statistics, and results of analysis of effects of other vs. female/male gender identity. N X columns indicate number of participants identifying as gender X (other or female/male). Score X columns show mean ± standard deviation (not standard error) for gender X. Rate X columns indicate rates of probable diagnosis for gender X. Slope is the fitted slope parameter from a linear model with time and age partialled out (see discussion of nuisance variables in the Materials and Methods). The slope parameter indicates the linear relationship between the other vs. female/male effect variable and the dependent variable, independent of time and age. P-values were derived from permutation testing on the slope parameters. Comparisons with an asterisk under the FDR column survived FDR multiple comparison correction. P-threshold for FDR correction was 0.025*.

**Table 7:** Effects of “prefer not to say” vs. female/male gender identity.

**Measure**	***N* female/male**	***N* preferred not to say**	**Score female/male**	**Score preferred not to say**	**Slope**	***P*-value**	**FDR**
CPSS score (PTSD)	8,391	200	13.00 ± 11.42	17.09 ± 13.66	4.11	0.00001	*
PHQ-A score (depression)	8,688	206	8.20 ± 6.49	10.25 ± 7.21	2.14	0.00001	*
HADS score (anxiety)	8,730	212	7.75 ± 4.69	9.11 ± 5.50	1.40	0.00004	*
CRAFFT score (alcohol/drugs)	8,768	214	0.58 ± 1.27	0.93 ± 1.62	0.41	0.00001	*
Rosenberg score (self-esteem)	8,662	216	18.06 ± 6.52	15.54 ± 6.87	−2.56	0.00001	*
Kidscreen score (quality of life)	8,711	215	26.83 ± 8.28	24.33 ± 8.97	−2.70	0.00001	*
CYRM-12 score (resilience)	8,540	203	46.95 ± 9.05	41.62 ± 10.59	−5.40	0.00001	*
**Measure**	***N*** **female/male**	***N*** **preferred not to say**	**Rate female/male %**	**Rate preferred not to say %**	**Slope**	***P*****-value**	**FDR**
Probable PTSD	8,391	200	38	51	12.88	0.00022	*
Probable depression	8,688	206	32	43	12.19	0.00024	*
Probable moderately severe depression	8,688	206	18	29	11.80	0.00003	*
Suicidal thinking	8,752	211	16	26	9.81	0.00018	*
Probable anxiety	8,730	212	28	43	15.35	0.00001	*
Probable alcohol/substance use disorder	8,768	214	16	25	10.66	0.00006	*
Tobacco use	8,792	217	13	19	7.44	0.0012	*
Any of 4 probable diagnoses	8,934	225	48	62	14.50	0.00003	*

*Table shows participant numbers, summary statistics, and results of analysis of effects of preferred not to say vs. female/male gender identity. N X columns indicate number of participants identifying as gender X (preferred not to say or female/male). Score X columns show mean ± standard deviation (not standard error) for gender X. Rate X columns indicate rates of probable diagnosis for gender X. Slope is the fitted slope parameter from a linear model with time and age partialled out (see discussion of nuisance variables in the Materials and Methods). The slope parameter indicates the linear relationship between the preferred not to say vs. female/male effect variable and the dependent variable, independent of time and age. P-values were derived from permutation testing on the slope parameters. Comparisons with an asterisk under the FDR column survived FDR multiple comparison correction. P-threshold for FDR correction was 0.025*.

## Discussion

This study investigated the multi-year impacts of wildfires on youth mental health. We examined 9,376 mental health survey samples from students in Grades 7–12 in Fort McMurray, Alberta. Surveys were conducted in 2017, 2018, and 2019, in the aftermath of the 2016 Fort McMurray wildfire.

As we have previously reported, the Fort McMurray student population exhibited elevated rates of probable depression, suicidal thinking, and tobacco use; elevated symptoms of anxiety; and reduced scores for quality of life and self-esteem 18 months after the 2016 wildfire, as compared to a control population that had not recently experienced a natural disaster (12). At that time, we observed similar mental health patterns for youth who were not actually present in Fort McMurray during the 2016 wildfire, although youth with greater personal exposure to impacts of the fire (e.g., home destroyed) exhibited worse symptoms of PTSD, depression, anxiety, and alcohol/substance use and lower scores for self-esteem and quality of life (13). The current study provides evidence of ongoing long-term mental health impacts on youth 3.5 years (42 months) post-wildfire.

### Longer Term Mental Health Impacts

The results from the present study indicate a slow but statistically significant trend of worsening mental health from 2017 to 2019, including increased symptom scores and increased rates of probable diagnoses of PTSD, depression, anxiety, drug use, and alcohol use. Quality of life and self-esteem scores also decreased from 2017 to 2019. Tobacco use and resilience scores did not change significantly. These findings are consistent with other studies reporting long-term negative impacts on mental health from natural disasters (41–45), although these studies are not directly comparable because they focused on mental health in adulthood and/or used different outcome measures.

Our results do not support our hypothesis that mental health would improve with time following the 2016 wildfire. One possibility is that 3.5 years may not be sufficient time for this population to recover from the adverse mental health effects of the wildfire, though that possibility seems unlikely in light of reports that many individuals affected by disaster do recover within 1–2 years (4). Theories of recovery from trauma identify various factors important to the recovery process including a sense of safety and stability, self- and community efficacy, hope, support from family and friends, social support, and social connectedness (4, 51, 52). It is possible that one or more factors important to recovery may be at issue. For example, Fort McMurray has experienced an economic downturn related to the reduction in oil prices starting in 2014, and this may negatively affect the community's sense of stability and hope. Future studies would be needed to test this suggestion.

It is noteworthy that CYRM-12 resilience measures did not change significantly with time (*p* = 0.27), indicating that the above-discussed changes in mental health measures may not be attributable to a change in resilience.

There was a statistically significant increase in suicidal thinking with age in the analysis of participants aged 11–19 (*p* = 0.0088) but not in the analysis of participants aged 12–17 (*p* = 0.11). The difference in results seems to be driven by the larger proportion of students aged 18 and 19 exhibiting suicidal thinking (18 years: 23%, 19 years: 28%) compared to younger students (14–19%) (see Table 4).

### Age-Related Differences in Mental Health Impact

We observed worse average scores on all 15 dependent measures in older vs. younger students. Specifically, older students exhibited higher mental health symptom scores, higher rates of probable diagnoses, and lower scores for self-esteem, quality of life, and resilience. These results are consistent with previous reports of increased mental health impairment among older youth compared to younger youth post-disaster (68), as well as higher rates of mental health symptoms in older adolescents more generally (69). As has been suggested by others (68), one possible interpretation of this finding, that would bear future exploration, is that greater awareness among older youth regarding challenges facing their families and the larger community (rebuilding, economic implications, etc.), as well as concerns they may have regarding their future, may negatively influence their well-being compared to younger youth. That older students also exhibited higher mental health symptom scores and lower resilience scores is consistent previous research showing an association between lesser resilience and worse mental health outcomes following disaster (28, 35, 70, 71) and with theoretical conceptions of resilience and its role in buffering the individual's mental health from harm due to adverse experiences (72–74). Reduced resilience may have played a role in making older students more vulnerable to developing negative mental health symptoms.

### Differences in Mental Health Related to Gender Identity

Our analyses revealed worse average scores on all 15 dependent measures in students identifying as female vs. male, in students with other gender identity vs. females/males, and students who preferred not to say their gender identity vs. females/males. These specific groups of students exhibited higher mental health symptom scores, higher rates of probable diagnoses, and lower scores for self-esteem, quality of life, and resilience.

We interpret participants answering “other” to the question of their gender identity as belonging to a gender minority, including transgender or gender non-conforming. For participants who preferred not to answer the demographics question on gender identity, some presumably identified as female or male but did not want to say so, while others presumably identified as a gender minority, including transgender or gender-non-conforming. Given that the group who preferred not to answer exhibited significantly worse mental health results than those identifying as female or male, we suspect that a majority of the group who preferred not to answer in fact belonged to a gender minority, including transgender or gender-non-conforming.

The finding of worse mental health scores in females compared to males is consistent with a previous report of higher rates of mental health symptoms post-natural disaster in females vs. males (75). We are not aware of any previous studies examining the impact of natural disaster on the mental health of gender minorities, including transgender and gender non-conforming individuals. It is a benefit of our population study approach, with its large sample size, that we are able to do so for the first time. More generally, our results are consistent with previous reports indicating higher rates of mental health symptoms in gender minorities, including transgender and gender-non-conforming individuals, both adults and youth (76–79). Resilience scores were lower for females vs. males and for gender minorities vs. females/males, which is consistent with previous studies showing an association between lesser resilience and worse mental health outcomes following disaster (28, 35, 70, 71). This suggests that reduced resilience may have been a factor in specific groups' developing more negative mental health symptoms.

### Implications

The destructive nature of disasters tends to attract funding from government and charities to address the immediate aftermath of a disaster. Our results provide an example of worsening mental health impacts in youth during the period 1.5–3.5 years following a wildfire disaster. This has occurred in the context of an ongoing, whole-of-community “build back better” approach to post-wildfire recovery in the area (80), as well as multiple challenges that have faced the community since the 2016 wildfire, including a downturn in the economy (Additional challenges include disastrous flooding following the 2020 spring ice breakup and the outbreak of the COVID-19 pandemic in 2020, though these occurred after the last survey data collection was completed in 2019). Our results underscore the need for multi-year funding, interventions, and policies to address not only the short-term physical damage but also the long-term negative mental health effects of natural disasters.

In addition to focusing on symptomatology, there is a need to investigate factors associated with post-disaster recovery processes, as well as efficacy of psychosocial strategies during later phases of recovery relative to early interventions, while also recognising complex and evolving, social-ecological post-disaster contexts. As an example, given that specific groups exhibited greater negative mental health effects, namely older youth, females, and gender minorities, interventions and policies may be more effective if they take into account developmental stage and gender identity with respect to mitigating these effects.

### Limitations

Our analysis included a large dataset of 9,376 survey responses. Conducting full clinical interviews with this large number of participants is not feasible, and so we used clinical measures based on self-report questionnaires, which is a limitation of the study. As noted above, in addition to the 2016 wildfire, Fort McMurray has experienced an economic downturn since 2014. The resulting job losses and financial impacts on families also likely had an effect on youth mental health in addition to the 2016 wildfire. We are not aware of any study specifically on the effects of Alberta's economic downturn on youth mental health, though one report found a negative mental health impact in adults (81). Additionally, given that this study utilised anonymous data, we were not able to identify longitudinal trends in individuals, only in groups.

## Conclusion

This study presents a cross-sectional statistical analysis of longitudinal mental health measurements in a population of Fort McMurray youth in Grades 7–12 during the period of 1.5–3.5 years following the 2016 Alberta wildfire. Findings indicate that there was a long-term trend of worsening mental health during that period. These observations support previous reports that youth and communities experience long-term mental health impacts following major natural disasters, such as wildfires. Our findings emphasise the need for multi-year funding and programs to support child and youth mental health in communities that have experienced such disasters.

## Author's Note

Some portions of the manuscript, in the Materials and Methods section, are based on similar material from two previous papers (12, 13) presenting findings from separate analyses of the data from Fort McMurray collected in 2017 only.

## Data Availability Statement

The raw data supporting the conclusions of this article will be made available by the authors, without undue reservation. Requests for data access should be made by email to the corresponding author.

## Ethics Statement

This study involving human participants was reviewed and approved by the University of Alberta's Health Research Ethics Board (ethics protocol number Pro00072669 approved June 26, 2017). Data were collected as part of the Fort McMurray Public and Catholic Schools' standard curriculum to evaluate their support programs. Written informed consent from the participants' legal guardian/next of kin was not required to participate in this study, in accordance with the national legislation and the institutional requirements. Parents/guardians were notified of the study 2 weeks prior and were given the opportunity to opt their child(ren) out of the study. Participants themselves were given the opportunity to opt out of the study, as was explained to them at the beginning of each study survey session.

## Author Contributions

MB, VA, AG, PB-M, JD, CM-H, JO, MM, SN, DK, and PS: study design. MM, SN, and DK: data collection. MB, HP, IC, and PS: analysis. MB, HP, AG, IC, PB-M, JD, CM-H, BL, and PS: manuscript preparation. All authors contributed to the article and approved the submitted version.

## Conflict of Interest

The authors declare that the research was conducted in the absence of any commercial or financial relationships that could be construed as a potential conflict of interest.

## Abbreviations

CPSS: Child PTSD Symptom Scale
CRAFFT: CRAFFT Questionnaire (proper name of the questionnaire is CRAFFT)
CYRM-12: Child and Youth Resilience Measure
EMPATHY: Empowering a Multimodal Pathway Towards Healthy Youth project
FDR: false discovery rate
HADS: Hospital Anxiety and Depression Scale
Kidscreen-10: Kidscreen Questionnaire
PHQ-A: The Patient Health Questionnaire, Adolescent version
PTSD: post-traumatic stress disorder
Rosenberg: Rosenberg Self-Esteem Scale.
