# Supplementary material for: Mental Health Symptoms Unexpectedly Increased in Students Aged 11–19 Years During the 3.5 Years After the 2016 Fort McMurray Wildfire: Findings From 9,376 Survey Responses
Source: Front Psychiatry. 2021 May 20;12:676256. doi: 10.3389/fpsyt.2021.676256 (PMC8172807; doi:10.3389/fpsyt.2021.676256)
Supplement: Supplementary file 1 [file Data_Sheet_1.DOCX]

**Appendix A: Survey Description Script**

The following script was read to participants at the start of each data collection session:

“Hello. It's very nice to meet all of you. Thank you for having us here and for your time.

Fort McMurray Public and Catholic Schools are doing a survey, and you are invited to take part in it today. The survey will help efforts that deal with the after effects of the 2016 Wildfire.

The survey will ask questions about your state of mind. Some of these are personal questions. But all your answers are anonymous. That means that your name will NOT be recorded in the survey. So whatever answers you give, no one can link them back to you - not your teachers, not your parents, and not the people running the survey.

The survey is done on computer. You can use the mouse or trackpad to answer the questions. Or you can use the arrows keys and Enter key. If there's a question you really don't want to answer, you can skip it. But please do try to answer all the questions. Please take the time to read each question carefully. Please do the survey on your own. Do not talk with each other about the questions. Do not look at someone else’s answers. The answers are personal information. Please just do your own questions and your own answers. Thanks.

Some students are dealing with the after effects of the Wildfire just fine, but some students need a little extra help. The survey is really important in enabling the schools to provide help for students who need it. The survey will allow us to know how big this issue is, and hopefully it will help with getting more money and resources for the schools if needed. We really appreciate everyone taking the time today.

You can choose to do the survey or not. If you don't do the survey, your teacher will assign other class work.

Does anyone have any questions?”

**Appendix B: Mental Health Support Programs**

The following mental health programs were put in place to support students in grades 7-9 by the Fort McMurray Public and Catholic Schools after the May 2016 wildfire: Health Program of Study, Student Wellness Action Team (SWAT), Beyond the Hurt Program, Fourth R, Healthy Relationships Training, CBT Group, Signs of Self Injury, Students Assisting Students (SAS), Tell Them From Me Survey (TTFM), Friends (group), Journey of Hope, Heart Math, Kids in the Know, Mindfulness.

The following mental health programs were put in place to support students in grades 10-12 by the Fort McMurray Public and Catholic Schools after the May 2016 wildfire: Healthy Relationships, Mental Health Curriculum Guide for High School, Material within Career and Life Management (CALM) Course, Student Wellness Action Team (SWAT), Students Assisting Students (SAS), High School Guidance Counsellors, Anxiety Presentation, Cognitive Behavior Therapy, PARTY Program, Heart of the Matter, Tell Them From Me Survey (TTFM), PEERS Program (Social Skills), Mindfulness.

**Appendix C: Exclusion Criteria**

Details of exclusion criterion 3: Five of the questions in the Rosenberg questionnaire (questions # 1, 3, 4, 7, 10) ask how much the participant agrees with positive statements about themselves, for example, “On the whole, I am satisfied with myself.” The other five Rosenberg questions (questions # 2, 5, 6, 8, 9) ask how much the participant agrees with negative statements about themselves, for example, “At times, I think I am no good at all.” For each participant, we created a positive and a negative Rosenberg score, ranging from 0 to 15, by adding their answers to the five positive questions or five negative questions (after flipping the negative questions), respectively. For both the positive and negative Rosenberg scores, a score of 0 indicated low self-esteem, and a score of 15 indicated high self-esteem. We excluded participants with a positive Rosenberg score of 12 or more and a negative Rosenberg score of 3 or less. We also excluded participants with a positive Rosenberg score of 3 or less and a negative Rosenberg score of 12 or more. Both of these exclusion scenarios were based on our judgment that the extreme disparity between the positive and negative Rosenberg scores indicated that the participant was not paying attention to the survey or did not understand the questions.

Details of exclusion criterion 4: Eight of the ten questions in the Kidscreen questionnaire (i.e. all questions except #3 and 4) ask the participant how much they agree with positive statements about themselves, such as "Thinking about the last week, have you physically felt fit and well?" The remaining two Kidscreen questions asked about negative aspects of the participant such as "Thinking about the last week, have you felt sad?" We created a positive Kidscreen score by adding their answers to the eight positive questions, yielding a score from 0 to 32. A 0 score indicated high quality of life, and a score of 0 indicated low quality of life. We excluded any participant exhibiting both a positive Kidscreen score of 26 or more and a positive Rosenberg score (see paragraph above) of 3 or less. We also excluded any participant exhibiting both a positive Kidscreen score of 6 or less and a positive Rosenberg score of 12 or more. Our reasoning was that such an extreme disparity between the positive Kidscreen and positive Rosenberg scores – which measure the somewhat related factors quality of life and self-esteem – indicated that the participant was not paying attention to the survey or did not understand the questions.

Details of exclusion criterion 5: In the HADS questionnaire, the answer choice order is reversed for two of the seven questions (namely, questions 4 and 5). We created non-reversed and reversed HADS scores by adding each participant's answers to the non-reversed questions (questions #1, 2, 3, 6, 7) or reversing and adding the answers to the reversed questions (#4, 5). The non-reversed score ranged from 0 to 15, and the reversed score ranged from 0 to 6. For both, a score of 0 indicated low anxiety, and a high score indicated high anxiety. We excluded participants with both a non-reversed HADS score of 14 or more and a reversed HADS score of 0. We also excluded participants with both a non-reversed HADS score of 2 or less and a reversed HADS score of 15. These two exclusion scenarios were based on our judgment that the extreme disparity between the non-reversed and reversed HADS scores indicated that the participant was not paying attention to the survey or did not understand the questions.
